# Muscarinic Receptors and Their Antagonists in COPD: Anti-Inflammatory and Antiremodeling Effects

**DOI:** 10.1155/2012/409580

**Published:** 2012-11-24

**Authors:** George Karakiulakis, Michael Roth

**Affiliations:** ^1^Department of Pharmacology, School of Medicine, Aristotle University of Thessaloniki, 54124 Thessaloniki, Greece; ^2^Pulmonary Cell Research-Pneumology, University Hospital Basel, 4031 Basel, Switzerland

## Abstract

Muscarinic receptors are expressed by most cell types and mediate cellular signaling of their natural ligand acetylcholine. Thereby, they control numerous central and peripheral physiological organ responses to neuronal activity. In the human lung, muscarinic receptors are predominantly expressed by smooth muscle cells, epithelial cells, and fibroblasts. Antimuscarinic agents are used for the treatment of chronic obstructive pulmonary disease and to a lesser extent for asthma. They are primarily used as bronchodilators, but it is now accepted that they are also associated with anti-inflammatory, antiproliferative, and antiremodeling effects. Remodeling of the small airways is a major pathology in COPD and impairs lung function through changes of the extracellular matrix. Glycosaminoglycans, particularly hyaluronic acid, and matrix metalloproteases are among extracellular matrix molecules that have been associated with tissue inflammation and remodeling in lung diseases, including chronic obstructive pulmonary disease and asthma. Since muscarinic receptors have been shown to influence the homeostasis of glycosaminoglycans and matrix metalloproteases, these molecules may be proved valuable endpoint targets in clinical studies for the pharmacological exploitation of the anti-inflammatory and antiremodeling effects of muscarinic inhibitors in the treatment of chronic obstructive pulmonary disease and asthma.

## 1. Muscarinic Receptors

The muscarinic receptors are metabotropic receptors that may be linked to plasma membrane K^+^ or Ca^2+^ ion channels [1, 2]. They belong to the superfamily of rhodopsin-like, seven transmembrane domains, single-glycoprotein receptors that are connected by intra- and extracellular loops. Muscarinic receptors initiate intracellular responses via interaction with GTP-binding proteins (G-proteins), although activation of other signaling molecules has been reported [1, 3, 4]. There are five subtypes of muscarinic receptors, referred to as M_1_ to M_5_, based on the order of their discovery, and according to the nomenclature proposed by Caulfield and Birdsall [5]. Muscarinic receptors are symbolized in the literature as “M_1_ mAChR,” “M_1_-mAChR,” “m1AChR,” or “mAChR1” for the M_1_ receptor. In this paper muscarinic receptor subtypes will be referred to as M_1_, M_2_, M_3_, M_4_, and M_5_, according to IUPHAR [6] and the MeSH Browser [7] of the National Library of Medicine of the National Institute of Health, USA.

Molecular cloning revealed that the five muscarinic receptors are encoded by separate intronless human genes. The muscarinic receptor gene sequences have significant homologies with other members of this large super-family and across mammalian species. The seven hydrophobic transmembrane domains of the muscarinic receptors are highly conserved with an average of 66% identity. In contrast, their intracellular loops are less conserved, with the third intracellular loop being particularly variable and accommodating the binding domain of receptor subtypes. Between the fifth and the sixth transmembrane regions, muscarinic receptors possess a large intracytoplasmic loop that exhibits high divergence between the different subtypes and is considered to be responsible for the G-protein-coupling selectivity [8–10] The name and gene location of the human M_1_ is on chromosome 11q13; M_2_ is on chromosome 7q31-35; M_3_ is on chromosome 1q43; M_4_ is on chromosome 11q12-112; M_5_ is on chromosome 15q26 [8, 9, 11].

## 2. Intracellular Signaling of Muscarinic Receptors

**Figure 1:**
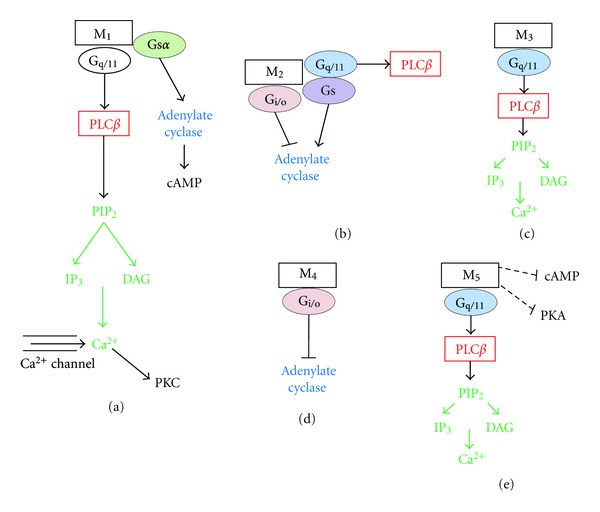
Receptor-specific G-protein coupling and signaling for the five human muscarinic receptors: (a) M_1_, (b) M_2_, (c) M_3_, (d) M_4_, and (e) M_5_.

As mentioned above, muscarinic receptors modulate different intracellular signal transduction pathways by coupling to multiple G proteins, which include stimulation of phospholipases C, A2 and D, cAMP degradation, cGMP production, attenuation of cAMP synthesis, and regulation of several ion channels [3, 10]. This diversity in signaling is more complicated, since a single muscarinic receptor subtype is capable of activating more than one type of G protein in a single cell and, thus, is coupled to more than one effector complements of the cell [3, 10, 12]. Muscarinic receptors can be divided into two groups according to their primary coupling efficiency to G-proteins. The first group of M_2_ and M_4_ muscarinic receptors couple to the pertusiss-toxin sensitive G_i/o_ type proteins. The second group including M_1_, M_3_, and M_5_ can couple to G_q/11_-type proteins [3, 5]. However, there is also evidence that muscarinic receptors couple to a wide range of signaling pathways, some of which are mediated by other types of G-proteins or other signaling mediators [13, 14]. An overview of known muscarinic receptor signaling is provided in Figure 1.

**Figure 2:**
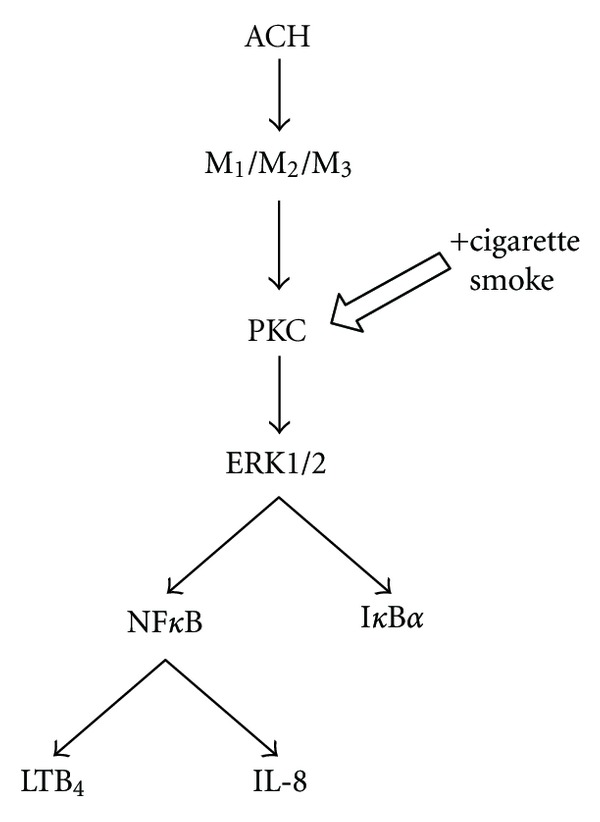
Synergistic effects of acetylcholine (ACH) and cigarette smoke on M_1_, M_2_, and M_3_ receptors. LTB4: leukotriene B4, PKC: protein kinase C, NF*κ*B: nuclear factor kappaB, and I*κ*B: inhibitor of NF*κ*B.

Studies on animal and human cell lines as well as on tissues demonstrated that muscarinic receptors also act via activation of the extracellular signal-regulated kinases 1 and 2 (ERK1/2) that is referred to as mitogen-activated protein (MAP) kinase 1 [15]. In human bronchial epithelial cells, it was demonstrated that various muscarinic receptor inhibitors including tiotropium (M_1_, M_2_, and M_3_ antagonist), gallamine (M_2_ antagonist), telenzepine (M_1_ antagonist), and 4-diphenylacetoxy-N-methylpiperidine methiodide (M_3_ antagonist) downregulated acetylcholine-induced leukotriene B_4_ release via the activation of ERK1/2 and nuclear factor-kappaB (NF*κ*B) pathways [16]. With respect to the involvement of muscarinic receptors in the regulation of inflammatory response, it has been reported that M_2_ and M_3_ receptors facilitate cigarette-smoke-extract-induced interleukin (IL)-8 secretion by in human airway smooth muscle cells via a protein kinase C-dependent activation of the inhibitor of I*κ*B*α* and ERK1/2 [17], which suggests a signaling pathway depicted in Figure 2.

## 3. Functional Role of Muscarinic Receptor Subtypes in the Lung

Muscarinic receptors are expressed by tissue-forming cells in the airways, predominantly by smooth muscle, epithelium, and fibroblasts. In the human lung, the density of parasympathetic cholinergic innervation is greatest in the proximal airways and diminishes peripherally. The predominant role of acetylcholine released by the parasympathetic system is in the control of distal airway resistance and the release of mucus from submucosal glands, and from goblet cells in the airway epithelium [18]. The distribution of muscarinic receptors in the human airway has been mapped by receptor autoradiography and in situ hybridization throughout the bronchial tree and is mainly restricted to muscarinic M_1_, M_2_, and M_3_ receptors [18–20], though M_4_ may also be involved. Acetylcholine released by cholinergic nerves regulates airway smooth muscle tone and mucus secretion [21]. 

In the human lung M_1_ subtype occurs not in the bronchus [20], but has been reported in human bronchial fibroblasts [22] and bronchial epithelial cells [16]. The presence of the M_1_ receptor mRNA was described in human peripheral lung tissue [19]. Stimulation of M_1_ receptors in the human lung causes bronchoconstriction and plays a modulatory role in electrolyte and water secretion [18, 23].

The presence of M_2_ receptors was reported in the human peripheral lung and the bronchus [20, 24]. Western blot analysis revealed the presence of M_2_ protein in human bronchial fibroblasts [22], epithelial cells [16], and smooth muscle cells [18]. Muscarinic M_2_ receptors are expressed by neurons, where they function as autoreceptors, limiting the release of acetylcholine from both preganglionic and parasympathetic nerve terminals of the lung [18, 21], of the human trachea [25], and of bronchi, but not of bronchioli [26]. Here, M_2_ mediated the inhibition of adenylyl cyclase and thereby preventing bronchodilation [27].

The M_3_ receptor is the primary muscarinic receptor subtype that mediates contraction of bronchial and tracheal smooth muscle, even though it is expressed in these tissues at considerable lower levels (about  1/4) than M_2_ [28]. M_3_ receptor is expressed by the smooth muscle cells of the airways [29], by human bronchial fibroblasts [22], and by human bronchial epithelial cells [16], as well as in the human peripheral lung [24]. The receptor predominantly occurs in the bronchus and its density decreases from the segmental to subsegmental bronchus and is abolished in lung parenchyma [20].

Stimulation of M_3_ receptors in the human lung, human central and peripheral airway smooth muscle, and in the human isolated bronchus causes bronchoconstriction and mucus secretion from submucosal glands [18, 27, 29–31]. However, activation of M_3_ receptors on vascular endothelial cells also induces the synthesis of nitric oxide, which diffuses to adjacent vascular smooth muscle cells and causes vasodilatation [32]. 

## 4. The Functional Role of Nonneuronal Muscarinic Receptor Subtypes in the Lung

During the past decade, several investigators have demonstrated that the biosynthesis, release mechanisms, and muscarinic receptors of the cholinergic system are functionally expressed independently of cholinergic innervations. It is concluded from such evidence that acetylcholine is not merely a neurotransmitter and that it transcends the nervous system, which in relation to lung pathophysiology can modify the phenotypic and cell function of airway cells, including epithelial cells (M_1_–M_4_), pulmonary vessel endothelial cells (M_1_–M_5_), mesenchymal cells, such as smooth muscle fibers (M_2_, M_3_) and fibroblasts (M_2_ > M_1_ > M_3_ > M_4_), and lung-infiltrating immune cells, such as mononuclear leukocytes (M_1_–M_5_) [33], monocytes, and macrophages (M_1_, M_2_, and M_3_) [34].

The function of nonneuronal acetylcholine released by the airway epithelium may participate in airway smooth-muscle contraction [35], but this remains controversial [36]. Additionally, acetylcholine, either neuronal or nonneuronal, may modulate airway inflammation and tissue remodeling [21]. For example, ensuing cellular effects in the airways following stimulation of M_1_ increased proliferation, while M_4_ activation increased migration and wound healing in epithelial cells. The stimulation of M_2_ increased proliferation of fibroblasts [33].

## 5. Muscarinic Receptors in Obstructive Pulmonary Diseases 

The pathophysiology of pulmonary obstructive diseases, such as chronic obstructive pulmonary disease (COPD) and asthma, is associated with the stimulation of the parasympathetic system, resulting in increased bronchoconstriction and mucus secretion from airway submucosal glands in the human lung. Since the early 70s, it has been established that it is the muscarinic receptor activity of acetylcholine that is involved in the pathophysiology of asthma and COPD. Muscarinic anticholinergic agents proved to be effective in the treatment of asthma and COPD, since the vagal cholinergic tone appears to be a reversible component of airway narrowing [18]. Thus, inhalation of ipratropium bromide, which inhibits M_1_, M_2_, and M_3_, was the first muscarinic inhibitor introduced for the treatment of patients with obstructive pulmonary diseases [37], followed by tiotropium bromide monohydrate that also binds to M_1_, M_2_, and M_3_ and has a longer duration of anticholinergic action [38]. Tiotropium has a considerably slower rate of dissociation from the M_1_ and the M_3_ receptors than from the M_2_ receptor, rendering kinetic selectivity of the drug for M_1_ and M_3_ receptors [39]. Thus, tiotropium is more effective, since it improves dyspnea and exercise capacity and reduces hyperinflation. It further reduces exacerbations in patients with moderate-to-severe COPD [40].

In addition, there is evidence from animal and human studies of defect expression and/or stimulation of muscarinic receptors in the lungs of asthma and COPD patients. It has been reported that M_2_ autoinhibitory receptors do not function normally in airways of some asthmatics [41]. The loss of function of M_2_ receptors mediated lung hyperreactivity in antigen-challenged animals and proposed to be an important cause of airway hyperreactivity in asthma [42]. The dysfunction of M_2_ autoinhibitory receptors in allergic asthma was proposed to be due to eosinophil-derived major basic protein, which acts as an allosteric antagonist of the M_2_ receptor [43], augmenting acetylcholine release, and this may modulate the cellular response associated with airway remodeling [44]. In leukocytes and the bronchi of patients with cystic fibrosis it was shown that the content of acetylcholine is substantially reduced, leading to reduced vesicle storage and transport of nonneuronal acetylcholine [33]. With respect to gene expression of muscarinic receptors, bronchoscopic evaluation of the mucosa in asthma patients revealed an increased expression of M_3_ receptor mRNA in severe asthmatics compared to patients with mild-to-moderate asthma and significantly higher levels of M_3_ receptor mRNA in patients with brittle asthma [45]. A similar investigation revealed that there are significantly lower levels of the M_3_ receptor mRNA in patients with COPD as compared to asthma patients, and that M_3_ receptor mRNA gene expression was significantly elevated in COPD patients with bronchial hyperresponsiveness as compared with patients without bronchial hyperresponsiveness [46], indicating that different molecular mechanisms underlie the clinical heterogeneity of bronchoconstriction in severe asthma and COPD. 

## 6. Muscarinic Receptors and Tissue Remodeling in the Lungs

Accumulating evidence over the past decade demonstrated that the pathology of asthma and COPD, in addition to bronchoconstriction, is attributed to inflammation of the airways [18]. The inflammation that occurs in asthma can be described as eosinophilic with an increase in Th2 (CD4^+^) cells, whereas inflammation that occurs in COPD is mainly neutrophilic with CD8^+^  T cells predominating [47]. Both neuronal or nonneuronal acetylcholine and muscarinic receptors appear to be involved in inflammation [21].

Pulmonary obstructive diseases are determined by cellular and structural changes of the airways, a process that was associated to chronic airway inflammation. Airway remodeling in asthma and COPD correlates with disease severity [48, 49] and is characterized by mucus gland hypertrophy, goblet cell hyperplasia, and pulmonary vascular remodeling [50]. Specific cellular and structural changes in asthma include basement membrane thickening, subepithelial fibrosis, and thickening of the airway smooth muscle bundle [51], while in COPD specific changes include peribronchial fibrosis and in severe stages of the disease increased airway smooth muscle mass [48]. Acetylcholine, neuronal or nonneuronal and muscarinic receptors appear to play an essential regulatory role in airway remodeling [21, 52, 53]. Recent studies in human-volunteering asthma patients, however, demonstrated that cholinergic stimuli and allergen can induce a very fast remodeling of the airway epithelium and the underlying mesenchymal cells within 8 days [53]. Interestingly, all features of remodeling were prevented by an inhaled beta2-agonist, leading the authors to postulate that relaxation of the bronchi prevented remodeling [53]. Based on our earlier studies, we suggest a more direct inhibitory effect of the beta2-agonist on various extracellular matrix genes [54].

Airway epithelial cells contribute to airway remodeling by hypersecretion of mucous and proliferation, while airway mesenchymal cells contribute by means of proliferation, expression of contractile protein, and the release of components such as mediators, extracellular matrix protein deposition, and matrix metalloproteinase (MMP) secretion [21, 55].

The hypersecretion of mucous by airway epithelial cells contributes to airway obstruction in chronic airway diseases [56]. In vitro and in vivo studies on animal models of asthma and COPD demonstrate the important role of acetylcholine in the regulation of mucus secretion [21]. Using human bronchus and cultured epithelial cells it was shown that the expression of MUC5AC is increased in asthma and COPD patients [57] and can be induced by carbachol and cigarette smoke extract while being inhibited by aclidinium, a long-acting muscarinic antagonist, or atropine [58]. Animals studies show that tiotropium inhibits increased MUC5AC expression and mucus gland hypertrophy in a guinea pig model of COPD [59], as well as the allergen-induced mucus gland hypertrophy and MUC5AC-positive goblet cell number [60]. Tiotropium also reduced the neutrophil elastase-induced goblet cell metaplasia in mice [61]. Acetylcholine may also regulate the proliferative and profibrotic response of airway epithelial cells, either through the induction of mechanical strain or by an autocrine/paracrine mechanism required for the repair of the damaged airway epithelium [21]. Epithelial cell proliferation and the expression of transforming growth factor (TGF)-*β* (profibrotic cytokine) were increased in bronchial biopsy specimens of patients with mild asthma following repeated challenge with methacholine or house dust mite allergen [53]. Animal studies indicated that acetylcholine induces proliferation of epithelial cells in the rat trachea, mediated by muscarinic M_1_ receptors [62] and of airway epithelial cells in monkeys [63].

**Figure 3:**
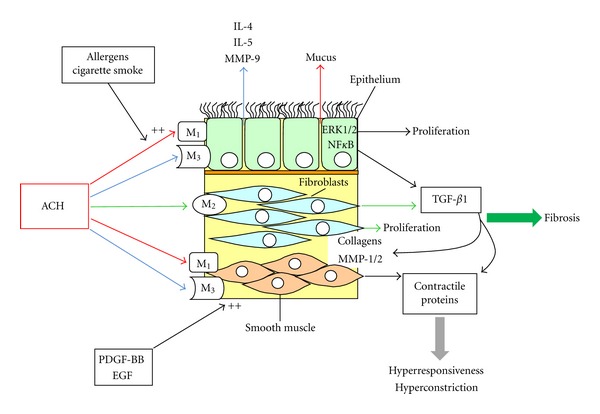
Cell type and muscarinic receptor specific effects on airway wall remodeling.

In the human lung, the stimulation of the M_2_ receptor induced cell proliferation of fibroblasts [44, 64] and acetylcholine enhanced cell proliferation in cells isolated from COPD patients, as compared to healthy nonsmokers, through a process involving ERK1/2 and NF*κ*B phosphorylation [65]. Airway smooth muscle thickening is a characteristic pathology of asthma, and to a lesser extent of COPD. Accumulating evidence suggests that stimulation of muscarinic receptors is involved in the proliferation and maturation of airway smooth muscle cells [21]. Furthermore, muscarinic receptor activation enhanced the mitogenic effect of platelet-derived growth factor (PDGF) and EGF on airway smooth muscle cells [66, 67]. However, the molecular interaction of the signalling cascades is not clear. Moreover, the expression of myosin light-chain kinase was augmented by carbachol in human airway smooth muscle cells exposed to cyclical mechanical strain [68] and stimulation of muscarinic receptors further enhanced the TGF-*β*1-induced expression of the contractile protein [69]. In animal models of asthma and COPD, tiotropium significantly inhibited airway smooth muscle remodeling and contractile protein expression in guineapigs [52, 60] and smooth muscle thickening and the expression of TGF-*β*1 in bronchoalveolar lavage fluid in an ovalbumine mouse model [70]. Similar effects have been described for the selective M_3_ receptor antagonist bencycloquidium bromide, which inhibited ovalbumin-induced mRNA expression of IL-5, IL-4, and MMP-9, as well as lung tissue eosinophil infiltration, airway mucus production, and collagen deposition in lung tissues in a murine asthma model [71]. The cell-type-specific expressions of muscarinic receptors and their effect on airway remodeling and inflammation is summarized in Figure 3.

## 7. Muscarinic Receptor and Extracellular Matrix Molecules

Extracellular matrix molecules, such as collagenous proteins, matrix metalloproteases (MMP), glycosaminoglycans (GAG), and proteoglycans play a key role in airway remodeling, inflammation, and emphysema [72–76]. 

### 7.1. Matrix Metalloproteases

Increased levels of MMP-1, MMP-2, and MMP-9 have been reported in the sputum [77] and lung parenchyma [78] of asthma or COPD patients. Hypoxia, which is associated with extracellular matrix remodeling in inflammatory lung diseases, such as fibrosis, COPD, and asthma, upregulated the expression of MMP-1, MMP-2, and MMP-9 precursors without subsequent activation in human lung fibroblasts and pulmonary vascular smooth muscle cells. MMP-13 expression was increased only in fibroblasts and PDGF-BB inhibited the synthesis and secretion of all hypoxia-induced MMP via ERK1/2 MAP kinase activation [73]. Same evidence indicates that muscarinic receptors mediate the expression of MMP in obstructive pulmonary diseases. Tiotropium inhibited TGF-*β*-induced expression of MMP-1 and MMP-2 in human lung fibroblasts, but had no effect on TGF-*β*-induced TIMP-1 and TIMP-2 expression [79, 80]. In contrast, bencycloquidium bromide, a selective M_3_ receptor antagonist, inhibited ovalbumin-induced expression of MMP-9 mRNA in a murine asthma model [71], indicating that M_1_ and M_3_ receptors mediate profibrotic and inflammatory response via specific MMPs. Evidence for the involvement of muscarinic receptors in the homeostasis of MMP comes also from other tissues. In human colon cancer, the activation of the M_3_ receptors stimulated the expression of MMP-1, MMP-7, and MMP-10, with subsequent transactivation of the epidermal growth factor receptor and proliferation [81].

### 7.2. Collagenous Proteins

Hypoxia and PDGF-BB induced synthesis of soluble collagen type I via ERK1/2 and p38 MAP kinase in human lung fibroblasts and pulmonary vascular smooth muscle cells [73]. In human lung fibroblasts stimulation of M_2_ receptors induced cell proliferation and collagen synthesis [44, 64]. In a clinical trial, inhalation of methacholine induced airway remodeling in asthma patients, through the expression of TGF-*β* and collagen type-I as shown in bronchial biopsies [53]. Treatment with tiotropium inhibited the increased peribronchial collagen deposition in a guinea pig COPD model [59].

### 7.3. Glycosaminoglycans (GAG)

GAG provide structural links between fibrous and cellular elements of the extracellular matrix. They contribute to viscoelastic properties, regulate permeability and retention of plasma components within the matrix, inhibit vascular cell growth, affect hemostasis, platelet aggregation, and interact with lipoproteins and various growth factors [82]. There are two main types of GAG: the nonsulphated hyaluronic acid and the sulphated GAG, heparan sulphate, heparin, chondroitin sulphate, dermatan sulphate, and keratan sulphate. With the exception of hyaluronic acid, GAG are usually covalently attached to a protein core, forming overall structures referred to as proteoglycans [82]. 

Evidence for the involvement of muscarinic receptors in the homeostasis of GAG comes from studies on various tissues, including the lung. In rat bladder, hyaluronic acid ameliorated H_2_O_2_-induced hyperactivity, possibly via the antioxidant activity and the inhibition of purinergic and muscarinic signaling pathway [83]. In rat vascular smooth muscle cells of the aorta, M_3_ receptors were involved in heparin-dependent relaxation [32]. In rabbits, acetylcholine-induced reactive oxygen species generation in myocytes and the intact heart was mediated via transactivation of EGF receptors through MMP-dependent release of heparin-binding EGF via muscarinic receptors [84]. In mouse pancreatic beta cells, heparin inhibited a muscarine-dependent ionic current [85]. In humans, inhaled heparin inhibited the bronchoconstriction induced by methacholine [86], even though contrary results have also been reported [87].

## 8. Conclusion

Muscarinic receptors and their intracellular molecular pathways comprise a major drug target in obstructive lung diseases. There is a need for further pharmacological exploitation of this crucial family of receptors as targets for more effective treatment of asthma and COPD. This huge potential transcends the beneficiary effect of antimuscarinic agents on bronchoconstriction and expands to anti-inflammatory, antiproliferative, and antiremodeling effects. Extracellular matrix molecules, such as GAG and MMP may be valuable biomarkers to determine the effect of muscarinic receptor inhibitors in clinical studies investigating drugs with anti inflammatory and anti-remodeling effects in the human lung.

## List of Abbreviations

COPD: Chronic obstructive pulmonary disease
EGF: Epidermal growth factor
ERK1/2: Extracellular signal-regulated kinases 1 and 2
GAG: Glycosaminoglycans
G-proteins: GTP-binding proteins
IL: Interleukin
MMP: Matrix metalloproteinases
M_1_, M_2_, M_3_, M4, and M_5_: Muscarinic receptors
NF*κ*B: Nuclear factor-kappaB
PDGF: Platelet-derived growth factor
TGF: Transforming growth factor.
